# Differentiating disseminated cryptococcosis from pulmonary cryptococcosis: roles of clinical characteristics and cryptococcal antigen

**DOI:** 10.1080/22221751.2026.2751728

**Published:** 2026-10-17

**Authors:** Xiaolan Zhou, Su Xu, Junhao Zhu, Qiangqiang Zhang, Min Zhu, Minggui Wang

**Affiliations:** a Huashan Worldwide Medical Center, Huashan Hospital, Fudan University, Shanghai, People’s Republic of China; b Institute of Antibiotics, Huashan Hospital, Fudan University, and Key Laboratory of Clinical Pharmacology of Antibiotics, National Health Commission of China, Shanghai, People’s Republic of China; c Department of Dermatology, Huashan Hospital, Fudan University, Shanghai, People’s Republic of China

**Keywords:** Cryptococcosis, dissemination, cryptococcal antigen, non-HIV, associated factors

## Abstract

Distinguishing disseminated cryptococcosis (DC) from localized pulmonary cryptococcosis (PC) is crucial due to divergent management and disparate prognoses. This study aimed to recognize associated factors for DC from PC in non-human immunodeficiency virus (HIV) patients. Accordingly, 475 pathologically confirmed cryptococcosis cases (391 PC and 84 DC) were retrospectively reviewed over a 20-year period at a tertiary hospital in China. Clinical, radiographic, dynamic serum cryptococcal antigen (CrAg) titer profiles, treatment and prognosis were analyzed. Patients with DC were significantly associated with a history of glucocorticoid therapy (16.7% vs. 5.6%; *P* *=* 0.001) and autoimmune diseases (16.7% vs. 6.1%; *P* *=* 0.001) compared with PC, aggressive thoracic computed tomography findings, including patchy exudation (*P* *=* 0.001) and cavitation (*P* *=* 0.009), and markedly higher baseline median serum CrAg titers (1:1280 vs. 1:80, *P* < 0.001). By multivariable analysis, CNS symptoms (adjusted OR [aOR] 131.12, 95% CI 10.21–1683.9, *P* *=* 0.001), chronic liver disease (aOR 15.21, 95% CI 1.06–217.69, *P* *=* 0.045), and baseline serum CrAg ≥1:80 (aOR 13.51, 95% CI 1.39–131.07, *P* *=* 0.025) remained independently associated with DC. An initial CrAg titer ≥ 1:80 should prompt careful CNS evaluation and consideration of lumbar puncture. Patients with DC had significantly longer median CrAg clearance (*P* *=* 0.001) and antifungal duration (18 vs. 6 months), with higher mortality (9.5% vs. 1.0%; *P* < 0.001) compared to PC. Early risk-stratification is imperative to optimize interventions and improve survival.

## Introduction

Cryptococcosis is a severe fungal disease caused primarily by the *Cryptococcus neoformans* and *Cryptococcus gattii* species complexes. Each year, an estimated 179,000 cases of HIV-associated cryptococcal antigenemia occur worldwide, leading to more than 112,000 deaths [1]. While traditionally characterized as an HIV-defining illness, the prevalence of this disease is increasing among the non-HIV-infected population. Recent studies from the United States and China reported similar results that over 50% of cryptococcosis cases occur in the non-HIV population [2–5]. Disseminated cryptococcosis (DC), particularly when it involves the central nervous system (CNS), is associated with a poor prognosis, with mortality rates ranging from 13.7% to 42.3% in the non-HIV population [6]. Beyond mortality, cryptococcal meningitis survivors may experience various long-term neurological and sensorial impairments, resulting in disability and poor quality of life [7]. Unlike routine CrAg screening is recommended in patients with HIV, validated tools for identifying non-HIV patients who require CNS evaluation remain limited [8, 9]. Current guidelines provide limited specific recommendations for non-HIV patients, and the decision to perform lumbar puncture (LP) in these patients is often based on clinical judgement rather than evidence-based criteria.

Pulmonary cryptococcosis (PC), characterized by localized infection within the lung parenchyma, is frequently encountered and often follows a subclinical course. In contrast, DC is defined by the involvement of two or more non-contiguous organ sites and most commonly manifests as cryptococcal meningitis or meningoencephalitis. The diagnosis of DC remains a formidable clinical challenge, primarily due to its protean nature and frequently atypical presentation. This diagnostic ambiguity is particularly critical because management strategies for PC and DC diverge substantially. Localized PC in immunocompetent hosts can often be managed with fluconazole monotherapy, while disseminated disease necessitates a more intensive, multi-phase regimen, typically involving amphotericin B formulations and flucytosine [8]. The delayed identification of DC precludes the timely initiation of standard-of-care, ultimately compromising patient prognosis. Despite the imperative for a definitive diagnosis, routine LP for all patients with suspected cryptococcosis is often impractical, particularly in resource-limited settings. Furthermore, the inherent risks of this invasive procedure must be meticulously weighed against its diagnostic yield. Consequently, robust, non-invasive risk-stratification tools are urgently needed. Identifying high-risk patients by synthesizing clinical symptoms, cryptococcal pathogen detection, and radiographic features is essential. Determining which subset of patients warrants invasive CSF evaluation remains a cornerstone of optimizing management and improving survival outcomes in patients with cryptococcosis.

Cryptococcal antigen (CrAg) testing is an important tool in both cryptococcosis diagnosis and monitoring. The European Organization for Research and Treatment of Cancer and the Mycoses Study Group Education and Research Consortium (EORTC/MSG) uses CrAg-positive cerebrospinal fluid and/or serum as evidence for proven cryptococcus [10]. Our early study demonstrated that CrAg titers declined during cryptococcosis therapy and this decrease correlated with fungal clearance. Therefore, CrAg titers may serve as a biomarker for monitoring treatment responses [11]. Although CrAg lateral flow assays (LFA) offer high sensitivity in patients with HIV, the serum CrAg positivity rate in non-HIV patients, especially those with localized pulmonary infections, is relatively low [6],leading to potential underdiagnosis. Previous studies reported that serum CrAg testing sensitivity could be as low as 82.6–90.9% in HIV-negative patients [12–14]. Consequently, histopathological examination remains the gold standard for definitive diagnosis. Special staining (e.g. periodic acid-Schiff, Grocott’s methenamine silver, and mucicarmine) of specimens obtained by percutaneous lung biopsy or surgical resection can provide definitive evidence of infection.

Huashan Hospital is a 3,000-bed tertiary teaching hospital in Shanghai, China. Its infectious disease discipline, comprising the Division of Infectious Diseases and the Institute of Antibiotics, serves as the National Medical Center for Infectious Diseases and maintains over 200 dedicated beds. With over 40 years of expertise in managing cryptococcosis and a CrAg testing programme established in the 1990s, the hospital serves as a major referral center for patients with fungal diseases in East China. In this study, we retrospectively analyzed pathologically confirmed cases over a 20-year period to delineate the clinical spectrum of cryptococcosis and identify associated factors of dissemination based on underlying diseases, clinical symptoms, radiologic findings, and CrAg titers, thereby providing evidence-based insights for early clinical intervention.

## Materials and methods

### Study design and participants

A retrospective epidemiological observational study was conducted in Huashan Hospital, Shanghai, China. A total of 475 inpatients meeting the following inclusion criteria and diagnosed with cryptococcosis during January 1, 1999, to December 31, 2019, were included. Medical data, including demographic characteristics, clinical symptoms, and laboratory and radiology results during hospitalization, were collected through the Hospital Information System. Treatment and laboratory data after discharge were obtained through telephone follow-ups. Immunosuppression was defined as receiving any dose of chemotherapy, including cyclophosphamide, mycophenolate mofetil, and methotrexate. Glucocorticoids use was defined as receiving any dose of glucocorticoids during the follow-up period.

### Inclusion and exclusion criteria

The inclusion criteria were: (1) hospitalized patients with a positive pathological diagnosis of cryptococci from lung tissue via surgical resection or biopsy or bronchoalveolar lavage fluid, and a negative serum HIV test result; (2) age ≥ 18 years; (3) complete medical history data and signed informed consent, and (4) no psychiatric disorders and the ability to communicate normally and complete telephone follow-up surveys. Informed consent and authorized delegation were obtained by phone in the presence of two study staff members.

The exclusion criteria were: (1) a clinical diagnosis of pulmonary cryptococcosis without pathological evidence, (2) a positive serum anti-HIV result, or the (3) refusal of the patient or their authorized representative to participate in the study.

### Diagnostic criteria

PC was defined as pathologically confirmed cryptococcal infection confined to the lungs, with no evidence of extrapulmonary involvement based on clinical evaluation, radiological findings, and laboratory testing. DC was defined as pathologically confirmed cryptococcosis involving two or more non-contiguous organ sites, regardless of pulmonary involvement. Patients presenting with concurrent pulmonary and extrapulmonary disease were systematically classified as having DC.

### Crag testing and CNS evaluation

Serum and CSF CrAg detection methods evolved during the study period. From 1999–2014, the latex agglutination test was used; since 2014, the LFA has been adopted. Both methods were performed according to the manufacturers’ instructions.

Cranial magnetic resonance imaging (MRI) and LP were not performed routinely for all patients. In practice, LP and cranial MRI were typically offered to patients who presented with elevated serum CrAg titers or neurological symptoms and provided consent. During follow-up, LP was reconsidered if patients developed new CNS symptoms or exhibited slower-than-expected CrAg titer decline. All decisions were made at the discretion of the attending physicians. LP specimens were routinely subjected to CSF CrAg testing, India ink staining, and fungal culture.

### Treatment efficacy evaluation

Therapeutic outcomes were evaluated at the end or upon discontinuation of antifungal therapy, based on clinical, radiological, and laboratory criteria. “Cure” was defined as the complete resolution of clinical symptoms. For DC patients with CNS involvement, “cure” was defined as complete resolution of clinical symptoms, radiological improvement or resolution on chest CT and/or cranial MRI, and negative CrAg tests in both serum and cerebrospinal fluid. For patients with PC, or DC without CNS involvement, CrAg test of cerebrospinal fluid is not included. “Improvement” was defined as meeting one or two of the three criteria (clinical, radiological, or laboratory) after treatment. “Treatment failure” was defined as the absence of clinical, radiological, or microbiological improvement, disease progression, or mortality during antifungal therapy. All-cause mortality during treatment was recorded and explicitly classified as treatment failure. For comparative analysis, outcomes of “cure” and “improvement” were combined into the clinically effective group, whereas cases classified as “treatment failure” or “death” were categorized into the treatment failure group.

### Ethical approval and trial registration

This study was approved by the Ethics Committee of Huashan Hospital, Fudan University (Approval No. 2020-653). The study was registered with the Chinese Clinical Trial Registry (ClinicalTrials.gov identifier: ChiCTR2000035805).

### Statistical analysis

Data analyses were performed using SAS software (version 9.3.3). Continuous variables with a normal distribution are presented as mean and standard deviation (SD) and were compared using Student’s t-test. Continuous variables with a skewed distribution are summarized as medians (interquartile range, IQR) and compared using the Mann–Whitney U test. Categorical variables are described as frequencies and percentages (%), with group differences assessed using the chi-squared test. The Cochran–Armitage test for trend was applied to evaluate trends in CrAg levels among patients with different types of cryptococcosis. The diagnostic performance of serum CrAg titer for distinguishing DC from PC was assessed using ROC analysis. ROC curves were generated with binary logistic regression using PROC LOGISTIC in SAS, modelling disease dissemination (event = disseminated) as a function of titer score. The area under the curve (AUC) with its 95% confidence interval was computed. The optimal titer cutoff was selected to maximize the Youden index (sensitivity + specificity − 1).

Multivariable logistic regression with backward elimination (entry *P* < 0.10, stay *P* < 0.05) was performed to identify variables independently associated with DC. Variables with *P* < 0.10 in univariable analysis were entered into the multivariable model. Odds ratios (ORs) with 95% confidence intervals (CIs) and *P* -values are reported.

The log-rank test was used to compare differences in the time to antigen titer negativity between patient groups with different types of cryptococcosis. Kaplan–Meier curves were generated to visualize and compare survival distributions. A *P*-value of < 0.05 was considered statistically significant.

## Results

### Baseline characteristics and geographic distribution

Among 475 patients with pathologically confirmed cryptococcosis, 391 (82.3%) were diagnosed with PC and 84 (17.7%) with DC. No patient initially classified with PC developed extrapulmonary dissemination during the follow-up period. In total, 261 (54.9%) patients were diagnosed through surgical procedures and 214 (45.1%) through non-surgical methods. Among the latter, 145 (67.8%) underwent percutaneous lung puncture biopsies, 15 (7.0%) underwent bronchoscopy biopsies, and 8 (3.7%) had cryptococcus isolated from bronchoalveolar lavage fluid or pleural effusion. The remaining 46 (21.5%) lacked detailed specimen acquisition records but were classified as non-surgical based on pathology reports and follow-up computed tomography (CT) findings of pulmonary nodules. The differences in baseline characteristics between PC and DC group were presented in Table 1.

**Table 1.** Demographic and clinical characteristics of invasive cryptococcosis patients.

	Total (n = 475)	Pulmonary cryptococcus (n = 391)	Disseminated cryptococcus (n = 84)	*P*-value
Mean age, yrs (SD)	49.7 (12.1)	49.9 (11.6)	48.8 (13.9)	0.450
Age group, n (%)				0.412
< 40 yrs	92 (19.4)	71 (18.2)	21 (25.0)	
40–50 yrs	159 (33.5)	134 (34.3)	25 (29.8)	
51–60 yrs	126 (26.5)	107 (27.4)	19 (22.6)	
> 60 yrs	98 (20.6)	79 (20.2)	19 (22.6)	
Male sex	304 (64.0)	251 (64.2)	53 (63.1)	0.849
Immunosuppressant use	30 (6.3)	22 (5.6)	8 (9.5)	0.183
Glucocorticoid use	36 (7.6)	22 (5.6)	14 (16.7)	0.001
Underlying disease, n (%)				
Malignancy	42 (8.8)	38 (9.7)	4 (4.8)	0.147
Chronic liver disease	40 (8.4)	29 (7.4)	11 (13.1)	0.089
Diabetes	39 (8.2)	35 (9.0)	4 (4.8)	0.204
Autoimmune disease	38 (8.0)	24 (6.1)	14 (16.7)	0.001
Biopsy method, n (%)				<0.001
Surgical resection	261 (54.9)	253 (64.7)	8 (9.5)	
Non-surgical biopsy	214 (45.1)	138 (35.3)	76 (90.5)	
Symptoms, n (%)	267 (56.2)	186 (47.6)	81 (96.4)	<0.001
Respiratory, n (%)	219 (46.1)	185 (47.3)	34 (40.5)	0.279
Central nervous system	71 (14.9)	7 (1.8)	64 (76.2)	<0.001
Radiology, n (%)^‡^				
Nodular mass	388 (90.4)	331 (92.2)	57 (81.4)	0.005
Patchy exudation	78 (18.2)	55 (15.3)	23 (32.9)	0.001
Cavity	28 (6.5)	18 (5.0)	10 (14.3)	0.009
Mix	71 (16.6)	49 (13.6)	22 (31.4)	0.001
Cranial MRI, n (%)	119 (25.1)	61 (15.6)	58 (69.0)	0.001
Meningeal enhancement	23 (19.3)	1 (1.6)	22 (37.9)	0.001
Intraparenchymal lesion	33 (27.7)	3 (4.9)	30 (51.7)	0.001

SD, standard deviation; yrs, years; MRI, magnetic resonance imaging.

‡n = 429.

Among 84 patients with DC, 77 (91.6%) had CNS involvement, including 44 with meningitis and 33 with meningoencephalitis. Three patients (3.6%) had bone involvement at different sites, two (2.4%) had concomitant cryptococcal pleurisy, one had combined meningeal and pleural involvement, and one had brain and bone lesions. Pathological specimens from DC patients were obtained more often by non-resected biopsy than those from the PC group (90.5%, 76/84 vs. 54.5%, 138/253; *P* < 0.001). Among the 475 cases, 460 were admitted to infectious disease wards. Geographically, most of the cases were clustered in Eastern and Central China, particularly Shanghai, Zhejiang, and Jiangsu (Supplementary Figure 1).

The mean age of the total cohort was 49.7 ± 12.1 years, and 64.0% (304/475) were male. No significant differences were observed between the PC and DC groups regarding mean age (*P* *=* 0.450), age distribution (*P* *=* 0.412), or sex (*P* *=* 0.849).

With respect to predisposing factors, univariable analysis showed that patients with DC were significantly more likely to have received glucocorticoid therapy (16.7% vs. 5.6%; *P* *=* 0.001) and have underlying autoimmune diseases (16.7% vs. 6.1%; *P* *=* 0.001) than those with PC. Other comorbidities, including malignancies (8.8%), chronic liver disease (8.4%), and diabetes (8.2%), were distributed similarly between the two groups (all *P* > 0.05).

### Clinical and radiographic presentations

Clinical symptoms were present in 96.4% (81/84) of the patients with DC, a rate significantly higher than the 47.6% (186/391) observed in the PC group (*P* < 0.001). Common clinical symptoms included fever (18.1%, 86/475), cough (38.5%, 183/475), sputum production (25.1%, 119/475), chest pain (7.6%, 36/475), chest tightness (8.4%, 40/475), and headache (13.3%, 63/475). Respiratory symptoms were reported in a similar proportion of patients in the PC and DC groups (47.3% [185/391] vs. 40.5% [34/84]; *P* *=* 0.279). In contrast, CNS symptoms were significantly more prevalent in the DC group than in the PC group (76.2% [64/84] vs. 1.8% [7/391]; *P* < 0.001). Among the 63 patients with headache, 96.83% (61/63) had DC with CNS involvement.

Radiographic patterns on thoracic CT also differed significantly between the groups. While nodular or mass-like lesions were the most common finding overall (90.4%), they were more frequent in PC than in DC (92.2% vs. 81.4%; *P* *=* 0.005). Patients with DC exhibited a significantly higher prevalence of aggressive radiographic features, including patchy exudation (32.9% vs. 15.3%; *P* *=* 0.001), cavitation (14.3% vs 5.0%; *P* *=* 0.009), and mixed patterns (31.4% vs. 13.6%; *P* *=* 0.001) (Table 1).

### Neurological involvement

Overall, LP was performed in 91.4% (434/475) of the cohort, with an overall cerebrospinal fluid (CSF) CrAg positivity rate of 18.0% (78/434). Specifically, LP completion rates were 89.8% (351/391) in the PC group and 98.8% (83/84) in the DC group. This high rate of LP screening among PC patients substantially minimizes the likelihood of misclassifying occult or subclinical CNS involvement.

Cranial MRI was performed in 25.1% (119/475) of the total cohort, with a significantly higher rate in the DC group (69.0% vs. 15.6%; *P* *=* 0.001). Among patients who underwent cranial MRI, those with DC demonstrated a markedly higher proportion of CNS involvement: 37.9% (22/58) showed meningeal enhancement and 51.7% (30/58) presented with intraparenchymal lesions, compared with only 1.6% and 4.9%, respectively, in the PC group (*P* *=* 0.001 for both) (Table 1).

### Baseline serum crag titers

Among the 475 patients, baseline serum CrAg titers (defined as the first measurement within one month of diagnosis) were available for 189 patients (142 PC, 47 DC). For the remaining patients, the first CrAg results were obtained after one month of diagnosis (81 PC, 36 DC), or no serum CrAg records were available during the treatment course (3 PC, 1 DC).

To avoid underestimating initial serum CrAg positivity due to postoperative seronegative conversion following complete surgical resection, we evaluated CrAg sensitivity exclusively within the non-surgical diagnostic group (n = 138; 94 PC and 44 DC), yielding a baseline sensitivity of 93.5% (129/138).

We subsequently analyzed initial CrAg titers in a combined sub-cohort of 189 patients (142 PC and 47 DC), comprising patients diagnosed by non-surgical biopsies (n = 138) and those retaining CrAg positivity after surgical resection (n = 51). The distribution of initial CrAg of DC and PC group were shown in Figure 1. Patients with DC exhibited significantly higher serum CrAg titers than those with PC (median [IQR]: 1:1280 [1:640–1:2560] vs. 1:80 [1:10–1:640], *P* < 0.001), despite wide titer distributions in both groups.

**Figure 1.**
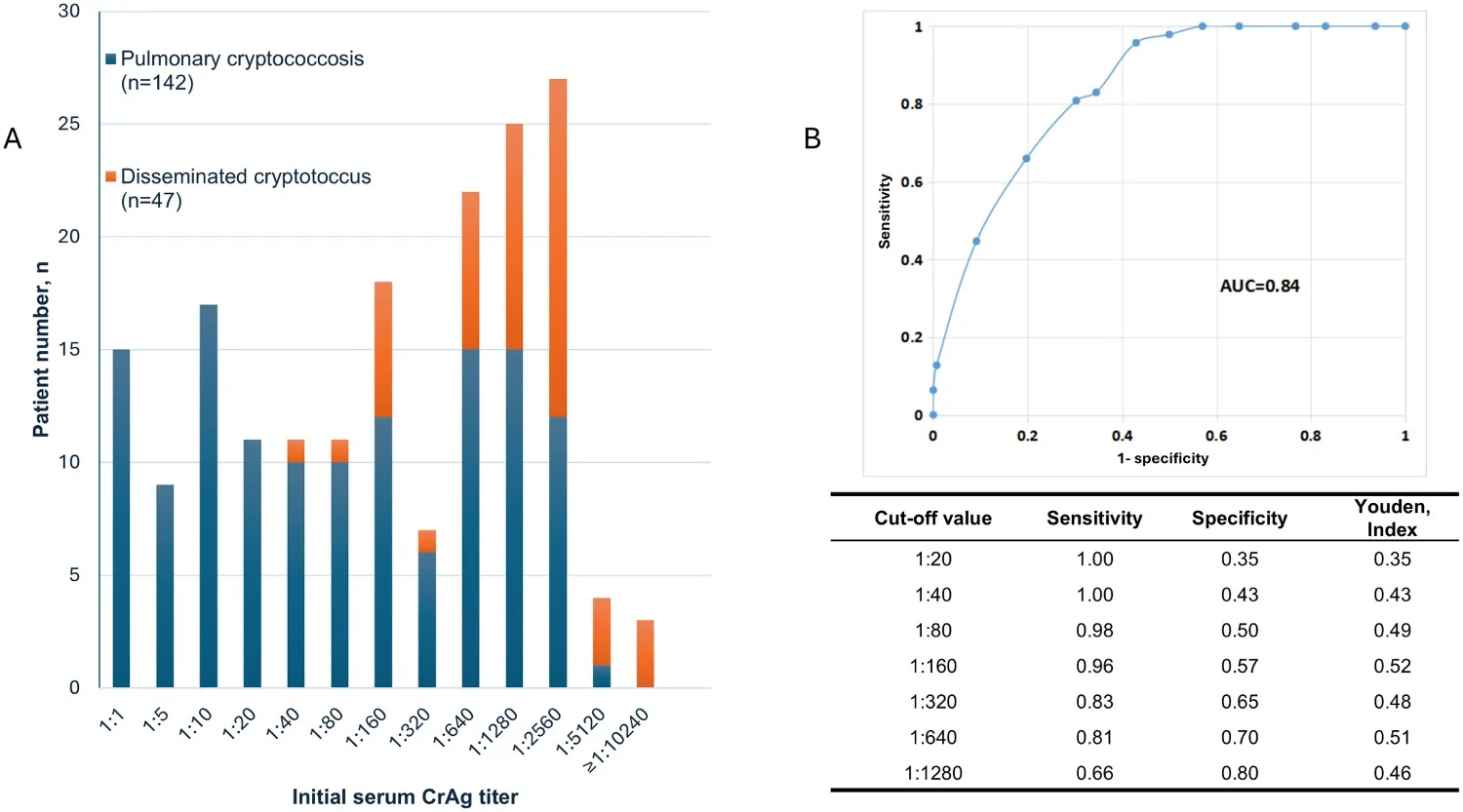
Comparison of baseline serum cryptococcal antigen (CrAg) titers between patients with pulmonary cryptococcosis (PC) and disseminated cryptococcosis (DC). (A) Distribution of baseline serum CrAg titers in PC and DC cohorts ($\chi^{2}=46.508,P<0.001$ by Chi-square test). (B) Receiver operating characteristic (ROC) curve analysis of baseline serum CrAg titers for discriminating DC from PC. At a cut-off threshold of ≥1:80, the sensitivity was 98% and specificity was 50% (Youden index = 0.49); at ≥1:160, the sensitivity was 96% and specificity was 57% (Youden index = 0.52). The ≥1:80 cut-off was selected for subsequent risk-stratification analyses to prioritize higher diagnostic sensitivity.

ROC analysis across these 189 baseline measurements demonstrated that the initial serum CrAg titer effectively identifies non-HIV patients at high risk of DC among those presenting with PC, yielding an area under the curve (AUC) of 0.838. A cut-off titer of ≥ 1:80 yielded a sensitivity of 97.9% and a specificity of 50.0%, whereas a cut-off of ≥ 1:160 provided a sensitivity of 95.7% and a specificity of 57.0%. High-level antigenemia (median titer ≥ 1:1280, range 1:80 to ≥ 1:1024) strongly characterized disseminated disease. Consequently, DC should be highly suspected when the initial serum CrAg titer is ≥ 1:80, and prompt LP with CSF analysis is recommended to rule out cryptococcal meningitis in these patients.

In the final multivariable analysis, three variables were independently associated with DC: CNS symptoms (adjusted OR [aOR] 131.12, 95% CI 10.21–1683.9, *P* *=* 0.001), chronic liver disease (adjusted OR 15.21, 95% CI 1.06–217.69, *P* *=* 0.045), and baseline serum CrAg ≥1:80 (adjusted OR 13.51, 95% CI 1.39–131.07, *P* *=* 0.025) (Supplementary Table 1).

### Species-level identification

Species-level identification was available for only a subset of patients via fungal culture, as most of the cohort was diagnosed through tissue biopsy, which precluded species identification. In total, 25 isolates derived from 23 patients were successfully cultured and preserved from this cohort (with two patients yielding two positive CSF cultures each during treatment). Among these preserved strains, only a single *Cryptococcus gattii* isolate was identified (from a CSF sample), whereas the remaining 24 isolates were confirmed as *Cryptococcus neoformans*.

### Serum crag dynamics during antifungal therapy

Continuous monitoring of serum cryptococcal antigen titers throughout the 25-month follow-up demonstrated divergent kinetic profiles between patients with PC and those with DC (Figure 2 and Supplement Table 2). The analysis comprised 388 patients with PC and 83 patients with DC, as serum CrAg records were unavailable for 3 patients with PC and 1 patient with DC.

**Figure 2.**
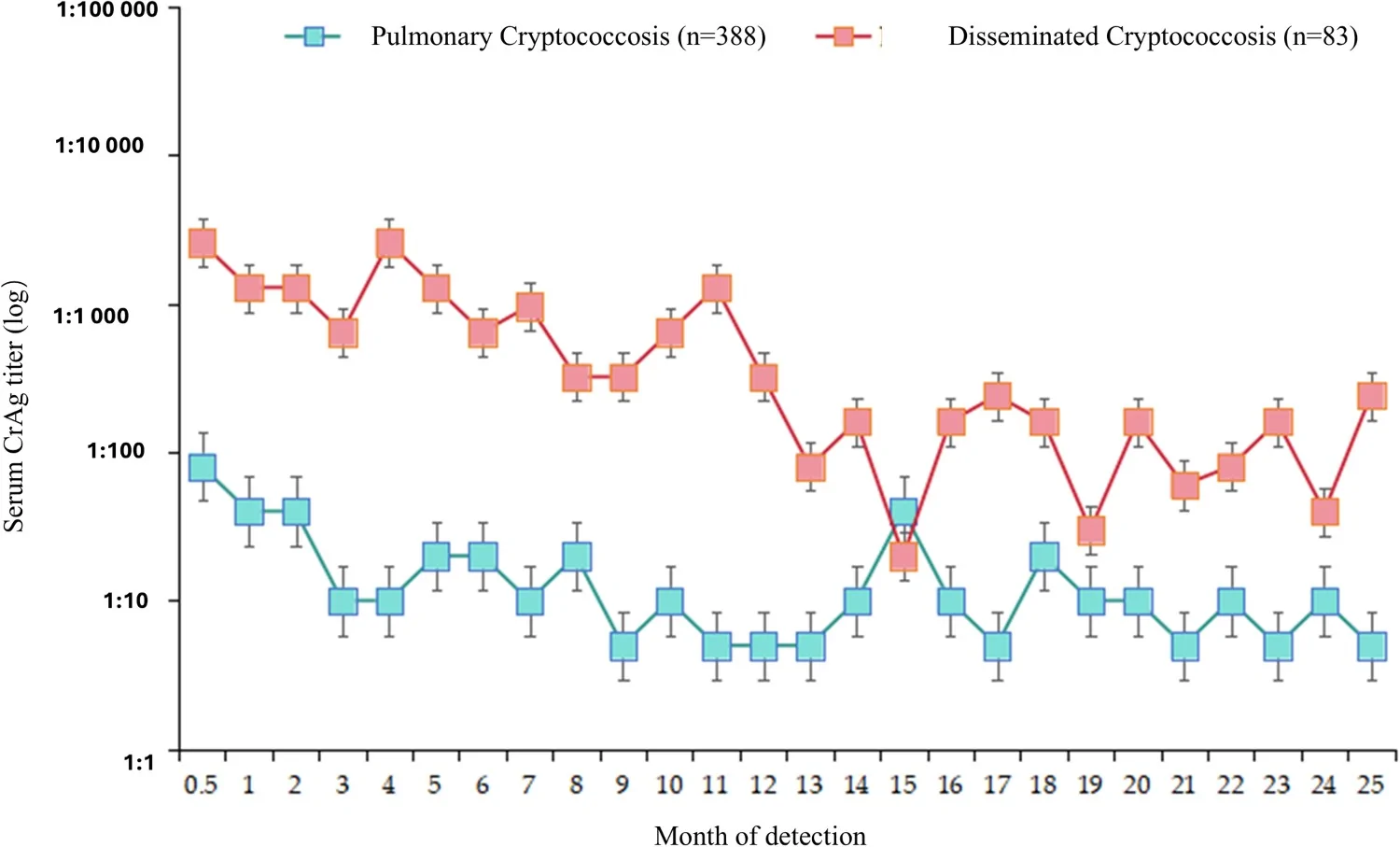
Longitudinal dynamics of serum cryptococcal antigen (CrAg) titers during antifungal therapy. Sequential serum CrAg titers were monitored across a 25-month follow-up period in patients with pulmonary cryptococcosis (PC, n = 388) and disseminated cryptococcosis (DC, n = 83). Patients with DC exhibited significantly higher baseline median titers and sustained elevated antigen levels throughout treatment compared to those with PC (*P* < 0.001). Data are presented as median values (P50) with interquartile ranges (IQR, P25–P75).

At the initial 0.5-month evaluation, the median (P50) serum CrAg titer in the DC group was 1:2560 (IQR, 1:1280–1:3840), significantly higher than the median titer of 1:80 (IQR, 1:10–1:1280) in the PC group. Although both groups exhibited an overall decline in fungal burden during the first 6 months of antifungal therapy, the DC group maintained substantially higher antigen loads. At 6 months, the median titer in patients with DC remained at 1:640 (IQR, 1:240–1:2560), whereas it decreased to 1:20 (IQR, 0 –1:160) in the PC group.

The divergence in CrAg clearance became more pronounced in the long-term follow-up. In the PC group, median titers remained consistently low after the seventh month of therapy (median range: 1:5–1:20). In contrast, the DC group displayed persistent antigenemia; even at the 24th month of treatment, the median titer in this group (1:40; IQR = 1:20–1:80) was four-fold higher than that of the PC group (1:10; IQR = 1:5–1:20). Notably, during the middle-to-late phases of therapy, occasional fluctuations in the P50 and P75 titers were observed in both groups, likely attributable to the drop-out of some patients with low titers who achieved antigen negativity by the end of the follow-up, thereby decreasing the total patient population.

### Dynamics of crag negative conversion

Kaplan–Meier analysis revealed significant differences in the time to conversion to serum CrAg negativity between infection types (Figure 3a; log-rank *P* *=* 0.001). This analysis was conducted on the same cohort shown in Figure 2. Among patients with PC, the median time to negative conversion was 23.7 months (95% confidence interval (CI): 20.9–26.5). On the contrary, patients with DC exhibited a markedly more protracted course of antigenemia, with less than half achieving seroconversion by the end of the 76-month follow-up. Throughout the follow-up period, the cumulative rate of negative conversion was significantly lower in the DC group than in the PC group (*P* *=* 0.001). At 24 months, a substantial proportion of patients with PC had achieved antigen clearance, but most patients with DC remained CrAg-positive, reflecting the high systemic fungal burden and slower clearance kinetics associated with disseminated disease.

**Figure 3.**
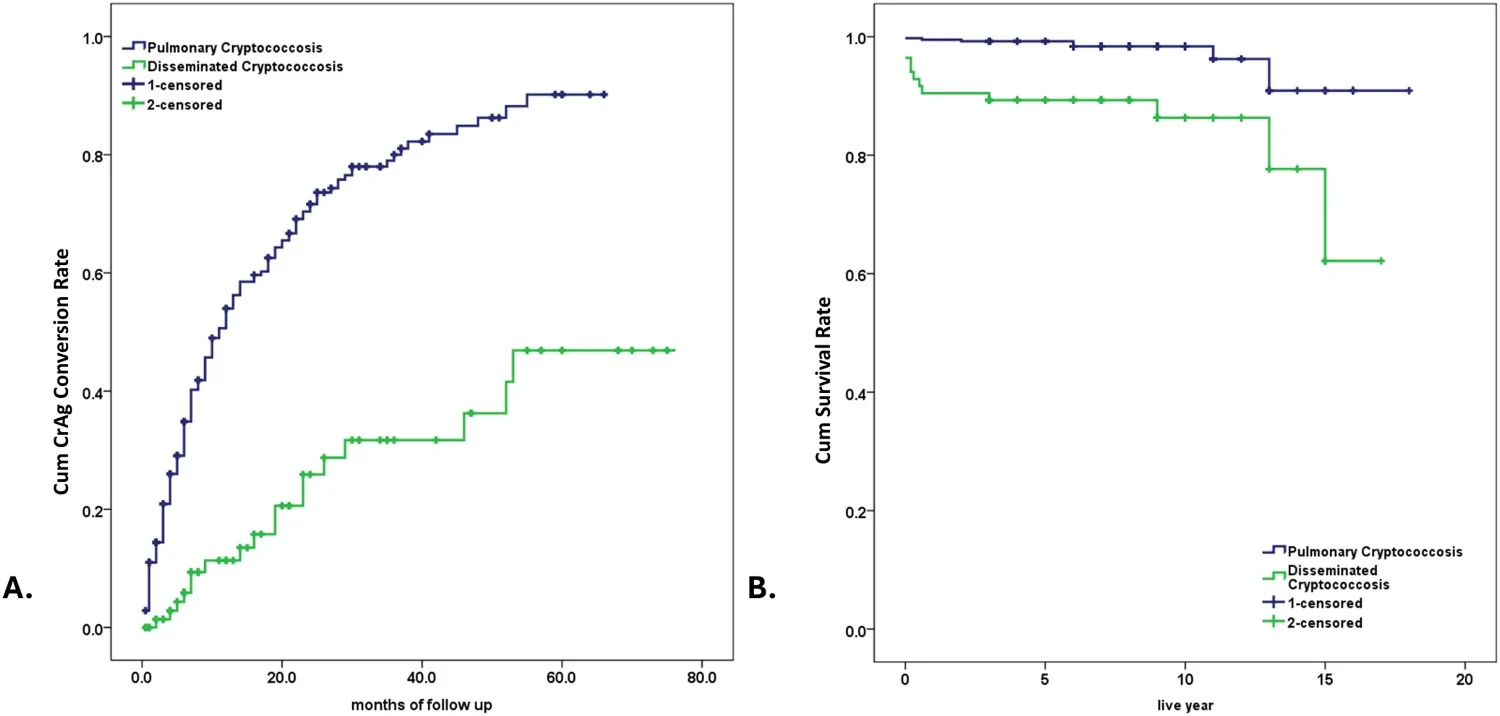
Kaplan–Meier estimates of antigen clearance and overall survival in patients with cryptococcosis. (A) Cumulative rates of serum cryptococcal antigen (CrAg) negative conversion in patients with PC (n = 210) versus DC (n = 77; log-rank test, *P =* 0.001). The median time to seroconversion in the PC group was 23.7 months, whereas most DC patients maintained persistent antigenemia throughout follow-up. (B) Overall survival curves for patients with PC (n = 391) and DC (n = 84; log-rank test, *P* < 0.001), demonstrating significantly higher mortality in the disseminated disease cohort.

### Antifungal therapy and clinical outcomes

Treatment strategies differed significantly by disease (Table 2). Triazole monotherapy was the primary induction regimen for patients with PC (77.2%), whereas 58.3% of patients with DC received amphotericin B-based regimens (*P* *=* 0.001). The median duration of antifungal therapy was 6 months (IQR, 3–12) for PC and 18 months (IQR, 12–36) for DC (*P* *=* 0.001). The overall cure rate was 66.5% (316/475), with a markedly higher rate in patients with PC than in those with DC (74.7% vs. 28.6%; *P* < 0.001). Mortality was significantly higher in the DC group than in the PC group (9.5% vs. 1.0%; *P* < 0.001). Kaplan–Meier survival analysis showed a significantly lower cumulative survival rate for patients with disseminated cryptococcosis (log-rank *P* < 0.001; Figure 3b).

**Table 2.** Treatment and outcome of cryptococcosis cases.

	Total (n = 475)	PC (n = 391)	DC (n = 84)	*P*
Induction antifungal regimen, n (%)				0.001
Triazole monotherapy	319 (67.2)	302 (77.2)	17 (20.2)	
Triazole plus 5-fluorocytosine	92 (19.3)	74 (18.9)	18 (21.4)	
Amphotericin B-based regimen	60 (12.6)	11 (2.8)	49 (58.3)	
None	4 (0.8)	4 (1.0)	0 (0.0)	
Duration of antifungal therapy, months, median (IQR)	6 (3–18)	6 (3–12)	18 (12–36)	0.001
Duration of antifungal therapy, n (%)				0.001
< 6 months	176 (37.05)	166 (42.5)	10 (11.9)	
≥ 6 months	299 (62.95)	225 (57.5)	74 (88.1)	
Outcome, n (%)				<0.001
Cured	316 (66.5)	292 (74.7)	24 (28.6)	
Improved	140 (29.5)	92 (23.5)	48 (57.1)	
Died	12 (2.5)	4 (1.0)	8 (9.5)	
Lost to follow-up	7 (1.5)	3 (0.8)	4 (4.8)	

PC: Pulmonary cryptococcosis.

DC: Disseminated cryptococcosis.

IQR: interquartile range.

Adverse events occurred in 6.6% (31/475) of the cohort. The DC group exhibited a higher incidence of adverse events than the PC group (16.7% vs. 4.4%; *P* *=* 0.001), with abnormal liver function test results (51.6%) and renal impairment (22.6%) being the most frequent adverse events (Table 3).

**Table 3.** Adverse events associated with different therapeutic regimens in patients with cryptococcosis.

Adverse event	Total (n = 475)	Pulmonary cryptococcosis (n = 391)	Disseminated cryptococcosis (n = 84)	*P*
Total incidence, n (%)	31 (6.6)	17 (4.4)	14 (16.7)	0.001
Type of adverse event, n (%)			0.045	
Headache	3 (9.7)	3 (17.6)	0 (0.0)	
Gastrointestinal distress	2 (6.5)	0 (0.0)	2 (14.3)	
Palpitation	1 (3.2)	1 (5.9)	0 (0.0)	
Abnormal liver function test	16 (51.6)	11 (64.7)	5 (35.7)	
Renal impairment	7 (22.6)	2 (11.8)	5 (35.7)	
Hypokalemia	2 (6.5)	0 (0.0)	2 (14.3)	

## Discussion

In this retrospective study, we identified key distinctions between PC and DC in non-HIV-infected patients, with particular emphasis on the clinically actionable indicators of dissemination. Our findings highlight two major observations: (1) radiographic and clinical features can provide clues to systemic spread and (2) serum CrAg titers offer substantial value for both risk stratification of dissemination and longitudinal disease monitoring.

Our study focused exclusively on non-HIV patients, a population in which decisions regarding CNS evaluation remain far less standardized compared to HIV-positive cohorts. In HIV-positive individuals, cryptococcal meningitis is driven predominantly by severe CD4+ T-lymphocyte depletion (typically < 100 cells/μL), for which routine serum CrAg screening and preemptive fluconazole therapy are well-established by international guidelines [8, 9]. In contrast, non-HIV populations exhibit highly heterogeneous immunologic backgrounds where specific comorbidities and distinct biomarker thresholds dictate the risk of disseminated disease. A study from Thailand underscored these underlying differences in patients with cryptococcal meningitis: among 46 HIV-uninfected individuals, 12 (26.1%) had received B-cell immunosuppressive therapy combined with corticosteroids, 5 (10.9%) had malignancies under targeted therapy, 3 (6.5%) presented with adult-onset immunodeficiency secondary to anti-IFN-γ autoantibodies, 1 (2.2%) was a solid organ transplant recipient, and 25 (54.3%) had no identifiable underlying conditions [14]. Whether the risk factors identified in our cohort – particularly the CrAg ≥ 1:80 threshold – apply equally to HIV-positive patients requires dedicated investigation, as antigen dynamics, fungal burden, and host immune responses differ substantially between the two populations. Ultimately, while existing literature on DC were mostly from HIV-positive cohorts, our study offers crucial complementary evidence by defining the distinct risk profiles and clinical characteristics within this understudied non-HIV population.

Conventional high-risk hosts of cryptococcosis include solid organ or hematopoietic stem cell transplant recipients, as well as patients receiving prolonged immunosuppressive therapy [8,15,16]. Recent studies have investigated various underlying risk factors for cryptococcosis [17]; however, few have focused on risk factors for disease progression. Tao et al. reported that underlying autoimmune disease, solid organ transplantation, and hematological malignancy are more common in extrapulmonary cryptococcosis than in PC [18]. Our data indicates that CNS symptoms, chronic liver disease and baseline CrAg ≥1:80 are significant independently associated with DC.

In our cohort, patients in the DC group were significantly more likely to be symptomatic than those in the PC group (96.4% vs. 47.6%). Radiographically, while nodular masses were common overall, DC was associated with more aggressive features on thoracic CT scans, including patchy exudation (32.9%), cavitation (14.3%), and mixed patterns (31.4%). A meta-analysis reported that CT findings of cavity signs, ground-glass attenuation, and mediastinal lymph node enlargement were significantly higher in immunocompromised patients [19]. Hu *et al*. reported that cavitation was more likely to occur in immunocompromised patients than in immunocompetent patients [20]. Beyond cavitation, few studies have reported that patchy exudation and mixed patterns in thoracic CT scans may be a sign of disease dissemination. Our findings indicate that these aggressive imaging features in non-HIV patients should prompt immediate evaluation for systemic dissemination, particularly involving the CNS. The fact that pathological specimens from DC patients were obtained significantly less often by surgical resection than those from PC patients’ group (9.5%, 8/84 vs. 45.5%, 115/253; *P* < 0.001) is likely related to the higher frequency of symptoms or typical radiology findings in the DC group, especially CNS symptoms and infiltrative lesions on chest CT.

In the final multivariable analysis, three variables were independently associated with DC: CNS symptoms, chronic liver disease, and baseline serum CrAg ≥1:80. Because dissemination events were limited (n = 84), the adjusted estimates have wide confidence intervals and should not be over-interpreted; our multivariable analysis is exploratory and associational rather than predictive, and while a baseline CrAg titer ≥1:80 marks increased risk of dissemination, establishing causation and the determinants of progression from pulmonary to disseminated cryptococcosis will require well-designed prospective cohorts.

Serum CrAg titers serve as a robust biomarker for both diagnosis and disease monitoring. In patients with HIV, the sensitivity and specificity of serum CrAg are reliable in diagnosing cryptococcosis. A meta-analysis reported serum CrAg sensitivity and specificity of 99.7% and 94.1%, respectively, in adults living with HIV [21]. However, the diagnostic efficiency is much lower in the non-HIV population. Hevey *et al.* reported serum CrAg LFA sensitivities of 82.6% in patients with disseminated disease and 90.9% in those with localized pulmonary disease [12]. Yang *et al.* reported that the sensitivity and specificity of the CrAg LFA test in 49 confirmed PC cases was 90.0% [13]. A retrospective multicenter study showed that negative serum CrAg results were significantly more frequent in patients with PC than in patients with DC [14]. In our study, the sensitivity of CrAg testing in biopsy-proven non-HIV patients was 93.5% (129/138), consistent with the previously published findings.

Previous reports showed that high CrAg titers are associated with disseminated disease. Dizon et al. evaluated 96 cases of cryptococcal disease, [32 each in low-titer (≤ 1:10), high-titer (≥ 1:20), and negative-titer groups]. PC was diagnosed in 9 (28%) low-titer and 8 (25%) high-titer patients (*P* *=* 1.00), and disseminated cryptococcosis occurred in 7 (22%) low-titer and 15 (47%) high-titers cases (*P* *=* 0.064) [22]. Baddley et al. reported that serum CrAg titers ≥ 1:64 were more common in patients with DC than in those with PC (71%, 17/24 vs. 22%, 17/79; *P* *=* 0.0001) [15].

To get the optimal cut-off serum CrAg titer for LP screening, we performed ROC analysis, as shown in Figure 1. While the 1:160 threshold yielded a marginally higher Youden Index (0.52 vs. 0.49), we deliberately prioritized higher diagnostic sensitivity (97.9% vs. 95.7%) in selecting 1:80. In clinical management, missing an occult CNS infection carries severe prognostic risks, including permanent neurological sequelae or death. A 1:80 cut-off effectively captures nearly all disseminated cases (missing <2.5%) while defining a targeted, clinically manageable subgroup of patients who warrant invasive lumbar puncture and mandatory CSF screening. Conversely, lowering the threshold further to 1:40 adds negligible diagnostic yield while unnecessarily exposing significantly more low-risk patients to invasive lumbar punctures. Thus, we recommend that a serum CrAg titer ≥1:80 should prompt careful CNS evaluation and consideration of lumbar puncture to exclude CNS. However, low CrAg titers have been reported in CNS cryptococcosis patients. Wang et al. reported that LP was performed in 97 patients with low serum CrAg titers (≤ 1:10), and positive CSF CrAg titers were reported in six patients [23].

Dynamic changes in serum CrAg levels in the patients were monitored throughout this study. Patients with DC maintained substantially higher fungal burdens throughout treatment. On the month 24, the median titer in the DC group (1:40) remained four-fold higher than that of the PC group (1:10). Although negative CrAg cannot be used as a drug withdrawal indicator, titer monitoring still has certain significance in determining therapeutic effects. The median time to CrAg negative conversion for patients with PC was 23.7 months. In contrast, patients with DC exhibited a markedly protracted course, with the majority remaining CrAg-positive after 2 years of therapy.

Over the 20-year study period, clinical practice has evolved substantially, including advancements in CrAg detection methods (transitioning from latex agglutination to LFA), broader adoption of high-resolution CT imaging, and expanded availability of antifungals. Nevertheless, first-line antifungal regimens in China remained relatively stable throughout most of the study timeframe. Notably, liposomal amphotericin B only became commercially available in China in 2023; thus, standard amphotericin B deoxycholate remained the primary formulation used across majority of our cohort. Meanwhile, the use of triazoles (primarily fluconazole and voriconazole) and flucytosine remained largely consistent over the past two decades. Although these long-term temporal shifts in diagnostics and therapeutic options could potentially confound our findings, the overall management framework remained comparable across historical sub-cohorts.

In the present study, favourable treatment outcomes (cure or improvement) were achieved in 98.2% of patients with PC and 85.7% of patients with DC. These findings are consistent with those of a recent study that reported that 89.0% of patients with PC recovered or improved after a 12-month follow-up period [24]. The therapeutic requirements for DC are significantly more intensive, with 58.3% of patients receiving amphotericin B-based regimens, whereas 77.2% of patients with PC were managed with triazole monotherapy. Despite more aggressive treatment, patients with DC had a significantly lower cure rate (28.6% vs. 74.7%) and higher mortality (9.5% vs. 1.0%). Furthermore, DC treatment was associated with a higher incidence of adverse reactions (16.7%), primarily renal impairment, and abnormal liver function. An in-depth large-scale cross-sectional observational study showed that combined flucytosine and polyene treatment was associated with lower 90-day mortality (adjusted odds ratio (aOR): 0.40 [0.23–0.71]; *P* *=* 0.002) in multivariable analysis and inverse probability of treatment weighted analysis (aOR: 0.45 [0.25–0.80]; *P* *=* 0.006) [25].

Notably, our findings have direct implications for clinical decision-making. Differentiating PC from DC is critical because the therapeutic strategies and prognostic trajectories differ substantially. Patients with DC required longer treatment durations, more intensive antifungal regimens, and experienced significantly higher mortality and adverse event rates. The observed delay in antigen clearance further reflects the persistent fungal burden and complexity of managing disseminated disease. Collectively, these findings underscore the importance of the early identification of high-risk patients who may benefit from prompt LP and initiation of aggressive antifungal therapy.

This study has several strengths. The requirement for histopathological confirmation minimizes misclassification and ensures a high degree of diagnostic certainty. Furthermore, the relatively large sample size allows for the comprehensive characterization of disease patterns in non-HIV-infected individuals, an increasingly important patient population. Several limitations should be acknowledged.

Several limitations of this study warrant consideration. First, its single-center, retrospective design inherently introduces potential selection and information biases, and clinical practices – including diagnostic imaging utilization and antifungal regimens – evolved over the 20-year study period. Second, restricting eligibility to pathologically confirmed cases may have introduced selection bias by omitting patients unsuitable for biopsy, thus, our findings may not fully generalize to the broader clinical spectrum. Third, although LP was performed in 89.8% of patients with PC and 98.8% of those with DC, CNS evaluation was not systematic in all patients at enrollment, and occult subclinical CNS involvement cannot be entirely excluded in the small minority of PC patients who did not undergo LP. Fourth, corticosteroid exposure was recorded without detailed dosing, duration, or timing, which precluded dose-stratified risk evaluations. Finally, species-level identification was culture-dependent and unavailable for patients diagnosed strictly by histology; although no *Cryptococcus gattii* was identified among the 341 archived isolates, its presence in culture-negative cases cannot be entirely excluded.

## Conclusion

In patients with non-HIV invasive cryptococcosis, the progression from localized PC to disseminated disease profoundly alters therapeutic regimens and prognosis. We identified glucocorticoid therapy, underlying autoimmune disease, and aggressive CT manifestations (patchy exudation and cavitation) as key associated factors of dissemination. We recommend a serum CrAg titer ≥1:80 should prompt careful CNS evaluation and consideration of lumbar puncture. DC typically requires a median of 18 months of intensive antifungal therapy and carries a significantly higher risk of treatment failure. These findings highlight the importance of early risk stratification to optimize antifungal stewardship and improve survival in this expanding patient population.

## Supplementary Material

Supplementary Table 2.docx

Supplementary Table 1.docx

Suppl Fig 1.png
