# Supplementary material for: Differentiating disseminated cryptococcosis from pulmonary cryptococcosis: roles of clinical characteristics and cryptococcal antigen
Source: Emerg Microbes Infect. 2026 Oct 17;15(1):2751728. doi: 10.1080/22221751.2026.2751728 (PMC13757930; doi:10.1080/22221751.2026.2751728)
Supplement: Supplementary file 1 [file TEMI_A_2751728_SM4966.docx]

**Supplementary Table 1. Logistic regression analysis of associated factors of disseminated cryptococcosis proportion in patients with cryptococcosis**

|  | **Proportion of DC,**  **n (%)** | **Univariable analysis** | | ***P*** | **Multivariable analysis** | | ***P*** |
| --- | --- | --- | --- | --- | --- | --- | --- |
|  |  | **ORc** | **95% CI** |  | **ORc** | **95% CI** |  |
| Age group, n (%) |  |  |  |  |  |  |  |
| <40 years | 21 (22.8) | ref |  |  | ref |  |  |
| 40-50 years | 25 (15.7) | 0.63 | 0.33-1.21 | 0.163 | 0.93 | 0.07-11.86 | 0.958 |
| 51-60 years | 19 (15.1) | 0.60 | 0.3-1.20 | 0.147 | 1.33 | 0.10-18.56 | 0.830 |
| >60 years | 19 (19.4) | 0.81 | 0.40-1.64 | 0.562 | 3.89 | 0.35-43.14 | 0.268 |
| **Gender, n (%)** |  |  |  |  |  |  |  |
| Male | 53 (17.4) | ref |  |  | ref |  |  |
| Female | 31 (18.1) | 1.05 | 0.64-1.71 | 0.849 | 3.22 | 0.32-32.90 | 0.324 |
| **Glucocorticoid therapy**  (yes vs no) | 38.9 vs 15.9 | 3.36 | 1.64-6.87 | 0.001 | - | - | - |
| **Underlying disease, n (%)** |  |  |  |  |  |  |  |
| Chronic liver disease  (yes vs no) | 27.5 vs 16.8 | 1.88 | 0.90-3.94 | 0.093 | **15.21** | **1.06-217.69** | **0.045** |
| Autoimmune disease  (yes vs no) | 36.8 vs 16.0 | 3.06 | 1.51-6.20 | 0.002 | - | - | - |
| **Symptoms**, n (%) | 30.3 vs 1.4 | 29.76 | 9.24-95.82 | 0.001 | - | - | - |
| Respiratory (yes vs no) | 15.5 vs 19.5 | 0.76 | 0.47-1.22 | 0.255 | - | - | - |
| Central nervous system  (yes vs no) | 90.1 vs 5.0 | 175.54 | 71.34-431.98 | 0.001 | **131.12** | **10.21-1683.9** | **0.001** |
| **Thoracic CT, n (%)** |  |  |  |  |  |  |  |
| Nodule/mass type  (yes vs no) | 14.7 vs 31.7 | 0.37 | 0.18-0.76 | 0.007 | 0.22 | 0.01-4.23 | 0.316 |
| Patchy exudation  (yes vs no) | 29.5 vs 13.4 | 2.71 | 1.52-4.81 | 0.001 | 0.60 | 0.04-9.26 | 0.714 |
| Cavitation (yes vs no) | 35.7 vs 15.0 | 3.16 | 1.39-7.17 | 0.006 | 2.09 | 0.09-49.79 | 0.649 |
| Mixed pattern (yes vs no) | 31.0 vs 13.4 | 2.90 | 1.61-5.22 | 0.001 | - | - | - |
| **Cranial MRI findings** |  |  |  |  |  |  |  |
| Meningeal enhancement (yes vs no) | 95.7 vs 37.5 | 36.67 | 4.74-283.7 | 0.001 | **3.98** | **0.08-207.03** | **0.003** |
| Intraparenchymal lesion (yes vs no) | 90.9 vs 32.6 | 20.71 | 5.82-73.73 | 0.001 | **6.51** | **0.39-107.61** | **0.001** |
| **Baseline serum CrAg titer** |  |  |  |  |  |  |  |
| <1:80 | 6 (2.0) | ref |  |  | ref |  |  |
| >=1:80 | 75 (44.4) | 38.96 | 16.43-92.39 | 0.001 | **13.51** | **1.39-131.07** | **0.025** |
