# Supplementary material for: Differentiating disseminated cryptococcosis from pulmonary cryptococcosis: roles of clinical characteristics and cryptococcal antigen
Source: Emerg Microbes Infect. 2026 Oct 17;15(1):2751728. doi: 10.1080/22221751.2026.2751728 (PMC13757930; doi:10.1080/22221751.2026.2751728)
Supplement: Supplementary file 2 [file TEMI_A_2751728_SM4967.docx]

**Supplementary Table 2. Comparison of CrAg titer changes in patients with pulmonary cryptococcosis or disseminated cryptococcosis**

| **months** | **PC (n=391)** | | | |  | **DC (n=84)** | | | |
| --- | --- | --- | --- | --- | --- | --- | --- | --- | --- |
|  | number | P25 | P50 | P75 |  | number | P25 | P50 | P75 |
| 0.5 | 31 | 10 | 80 | 1280 |  | 16 | 1280 | 2560 | 3840 |
| 1 | 77 | 1 | 40 | 640 |  | 36 | 640 | 1280 | 2560 |
| 2 | 75 | 5 | 40 | 320 |  | 42 | 320 | 1280 | 2560 |
| 3 | 66 | 1 | 10 | 160 |  | 39 | 160 | 640 | 2560 |
| 4 | 50 | 1 | 10 | 160 |  | 37 | 160 | 2560 | 2560 |
| 5 | 39 | 1 | 20 | 320 |  | 35 | 160 | 1280 | 2560 |
| 6 | 49 | 0 | 20 | 160 |  | 32 | 240 | 640 | 2560 |
| 7 | 36 | 0 | 10 | 60 |  | 22 | 80 | 960 | 2560 |
| 8 | 27 | 1 | 20 | 80 |  | 27 | 40 | 320 | 1280 |
| 9 | 31 | 1 | 5 | 40 |  | 17 | 40 | 320 | 2560 |
| 10 | 37 | 1 | 10 | 40 |  | 14 | 160 | 640 | 1280 |
| 11 | 27 | 1 | 5 | 40 |  | 11 | 160 | 1280 | 5120 |
| 12 | 33 | 1 | 5 | 40 |  | 15 | 10 | 320 | 640 |
| 13 | 22 | 1 | 5 | 20 |  | 11 | 5 | 80 | 640 |
| 14 | 23 | 1 | 10 | 40 |  | 11 | 10 | 160 | 1280 |
| 15 | 13 | 5 | 40 | 80 |  | 13 | 10 | 20 | 320 |
| 16 | 20 | 1 | 10 | 30 |  | 11 | 1 | 160 | 640 |
| 17 | 21 | 1 | 5 | 40 |  | 10 | 10 | 240 | 320 |
| 18 | 21 | 1 | 20 | 80 |  | 9 | 40 | 160 | 640 |
| 19 | 20 | 1 | 10 | 20 |  | 8 | 0.5 | 30 | 1360 |
| 20 | 15 | 2 | 10 | 20 |  | 9 | 40 | 160 | 640 |
| 21 | 11 | 1 | 5 | 20 |  | 8 | 15 | 60 | 160 |
| 22 | 17 | 1 | 10 | 20 |  | 5 | 20 | 80 | 640 |
| 23 | 11 | 1 | 5 | 20 |  | 7 | 0 | 160 | 640 |
| 24 | 14 | 5 | 10 | 20 |  | 7 | 20 | 40 | 80 |
| 25 | 17 | 1 | 5 | 10 |  | 4 | 120 | 240 | 800 |
